# Glycolysis in the tumor microenvironment: a driver of cancer progression and a promising therapeutic target

**DOI:** 10.3389/fcell.2024.1416472

**Published:** 2024-06-12

**Authors:** Junpeng Zhao, Dandan Jin, Mengxiang Huang, Jie Ji, Xuebing Xu, Fei Wang, Lirong Zhou, Baijun Bao, Feng Jiang, Weisong Xu, Xiaomin Lu, Mingbing Xiao

**Affiliations:** ^1^ Department of Gastroenterology, Affiliated Hospital of Nantong University, Medical School of Nantong University, Nantong, Jiangsu, China; ^2^ Department of Laboratory Medicine, Affiliated Hospital and Medical School of Nantong University, Nantong, Jiangsu, China; ^3^ Department of Clinical Medicine, Medical School of Nantong University, Nantong, Jiangsu, China; ^4^ Department of Gastroenterology, Affiliated Nantong Rehabilitation Hospital of Nantong University, Nantong, Jiangsu, China; ^5^ Department of Oncology Affiliated Haian Hospital of Nantong University, Nantong, Jiangsu, China

**Keywords:** tumor microenvironment, aerobic glycolysis, metabolic reprogramming, cancer progression, targeted therapy

## Abstract

Even with sufficient oxygen, tumor cells use glycolysis to obtain the energy and macromolecules they require to multiply, once thought to be a characteristic of tumor cells known as the “Warburg effect”. In fact, throughout the process of carcinogenesis, immune cells and stromal cells, two major cellular constituents of the tumor microenvironment (TME), also undergo thorough metabolic reprogramming, which is typified by increased glycolysis. In this review, we provide a full-scale review of the glycolytic remodeling of several types of TME cells and show how these TME cells behave in the acidic milieu created by glucose shortage and lactate accumulation as a result of increased tumor glycolysis. Notably, we provide an overview of putative targets and inhibitors of glycolysis along with the viability of using glycolysis inhibitors in combination with immunotherapy and chemotherapy. Understanding the glycolytic situations in diverse cells within the tumor immunological milieu will aid in the creation of subsequent treatment plans.

## 1 Introduction

In healthy cells, the main energy source is the mitochondrial oxidative phosphorylation reaction (OXPHOS). In contrast to normal cells, tumor cells choose glycolysis as a source of energy, even in well-oxygenated environments. This process is referred to as the “Warburg effect”, or aerobic glycolysis or metabolic reprogramming (Hanahan and Weinberg, 2011). Tumors are regarded as proliferative diseases of cells that are detached from the normal growth state and need more energy to achieve self-differentiation and highly active biological behavior; consequently, one of the key characteristics of tumors is the disruption of cellular energy metabolism. In addition to dysregulated energy metabolism, aerobic glycolysis is a crucial indicator of malignant transformation in tumor cells (Wang et al., 2023).

The TME is a highly ordered ecosystem mostly composed of tumor cells, extracellular matrix, and a variety of noncancerous cells, including immunological and stromal cells (de Visser and Joyce, 2023). Glycolysis not only promotes tumor growth but also modifies the TME by impacting immune cell and stromal cell development, activation, and function. Nonmalignant cells, such as immune cells (Kirchmair et al., 2023) and cancer-associated fibroblasts (Yan et al., 2018), undergo metabolic reprogramming during carcinogenesis that is notable for its increased dependence on glycolysis. Additionally, by promoting the production of metabolites and signaling chemicals, glycolysis of tumor cells can positively alter the TME (Guo et al., 2022; Liu et al., 2023). Antitumor immune cells, including CD4^+^ T cells, CD8^+^ T cells, and dendritic cells (DCs), are inhibited from absorbing enough glucose for their own glycolysis by extracellular acidosis. This leads to substrate competition and a reduction in antitumor activity (Krawczyk et al., 2010; Michalek et al., 2011). Tumor-associated macrophages (TAMs) and myeloid-derived suppressor cells (MDSCs), on the other hand, are protumor immune cells that continuously suppress the tumor immune response and are more tolerant of high lactic acid levels in the environment (Jian et al., 2017; Zhang D. et al., 2019).

Targeting pathways associated with glycolysis presents novel treatment prospects for malignancies, given the critical role that glycolysis plays in the progression of cancers. According to a substantial body of research, targeting important proteins in tumor cells that are involved in glucose uptake, glycolysis, lactate generation, and transport may have a substantial therapeutic impact on a number of malignancies. Furthermore, controlling metabolic reprogramming of the immune system and stromal cells opens up new avenues for anticancer treatment. This review aims to clarify the glycolytic environment in tumor cells and TME cells and to enumerate potential targets, offering suggestions for additional studies and potential therapeutic uses of targeted glycolysis in cancers.

## 2 Why is there a preference for glycolysis?

### 2.1 Advantages provided by glycolysis

Only two molecules of ATP are produced by the oxidation of glucose to pyruvate, whereas 31 to 38 molecules of ATP can be produced by complete oxidation to CO_2_ by oxidative phosphorylation. Although aerobic glycolysis is a less efficient producer of ATP than OXPHOS, rapidly proliferating tumor cells, as well as other cells dependent on the “Warburg effect”, are still able to maintain ATP levels similar to those of normal cells (Barba et al., 2024).

To elucidate why cancer cells employ aerobic glycolysis despite its obvious energetic disadvantage, it has been argued that the idea that OXPHOS produces energy more efficiently than aerobic glycolysis may not be plausible, or at least that the idea has a premise. Do not take into account the time required for the reaction, the breakdown of 1 mol of glucose by OXPHOS theoretically produces more ATP than does anaerobic glycolysis. For tumor cells, however, the availability of sufficient energy in a relatively short period of time is critical. It has been suggested that glycolysis produces energy faster than OXPHOS. This means that aerobic glycolysis produces more energy per unit time than OXPHOS under a steady supply of glucose (Pfeiffer et al., 2001).

Another view emphasizes that for actively proliferating tumor cells, meeting the energy demand is only one of the necessary aspects to ensure their biological activity. As cell division requires the consumption of massive amounts of biomolecules, tumor cells need to maintain a minimum energy supply based on ensuring the synthesis of biomolecules required for proliferation. Based on this view, it is understandable that tumor cells choose to inhibit OXPHOS in favor of aerobic glycolysis. Because the intermediates of the glycolytic process can be used as substrates for the synthesis of biomolecules required for proliferation, such as nicotinamide adenine dinucleotide phosphate (NADPH), ribose phosphate, and a number of nonessential amino acids, these biomolecules are used by tumor cells as raw materials for the synthesis of nucleic acids, lipids, and proteins (Vander Heiden et al., 2009).

### 2.2 The state of glycolysis in tumors

**FIGURE 1:**
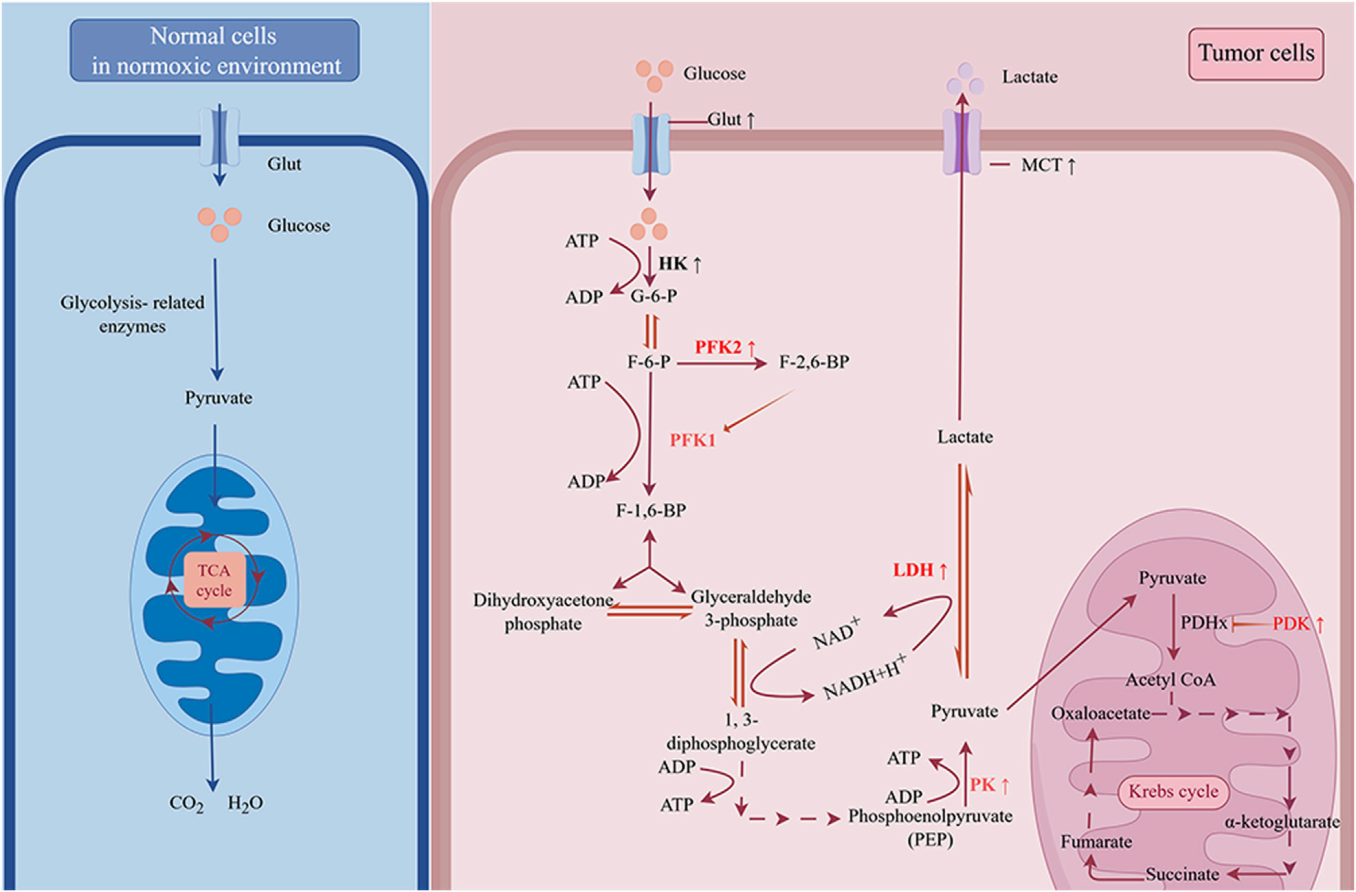
The “Warburg effect” in cancer. Under normoxic conditions, cells take up glucose and oxidize it to CO_2_ and H_2_O through glycolysis and oxidative phosphorylation (OXPHOS). However, for tumor cells, even in the presence of sufficient oxygen, glycolysis is the primary way to produce energy. The upregulation of glucose transporter (Glut) increases the ability of tumor cells to compete for glucose. By upregulating key enzymes involved in the glycolytic process, commonly including hexokinase (HK), phosphofructokinase 2 (PFK2), and pyruvate kinase (PK), tumor cells rapidly breakdown glucose into pyruvate. The accumulated pyruvate can be simply reduced to lactate by lactate dehydrogenase (LDH) and transported out of the cell by monocarboxylate transporter (MCT), or it can enter the mitochondria, where it is catalyzed by pyruvate dehydrogenase (PDH) to produce acetyl-CoA, which enters into a complex oxidative pathway for complete oxidation to CO_2_ and H_2_O. Pyruvate dehydrogenase kinase (PDK) inhibits the activity of PDH. The upregulation of PDK, LDH and MCT restricts the entry of pyruvate into the tricarboxylic acid cycle, resulting in greater conversion to lactate.

Glucose is the most significant energy source in the human body. The glucose transporter (Glut) transports glucose into the cell, where it is then transformed by a sequence of glycolytic enzymes into pyruvate, which is subsequently processed into lactate by lactate dehydrogenase (LDH). Eventually, the monocarboxylic acid transporter (MCT) takes the produced lactate outside of the cell, causing the cell to consume a significant amount of glucose and secrete a large amount of lactate. In tumors, these steps of glycolysis, including glucose uptake, the glycolytic process (from glucose to pyruvate), and lactate production and transport, are generally enhanced to sustain biological progression, as described in more detail below (Figure 1).

#### 2.2.1 Glucose uptake

Glucose uptake requires the assistance of the glucose transporter protein (Glut), and 14 kinds of Glut proteins have been identified, among which Glut1 serves as the primary carrier for glucose uptake in cells. In addition, the subcellular localization of Glut, especially its placement on the membrane, is critical for determining whether highly expressed Glut within cells can perform its transport functions (Zhang et al., 2021). By regulating the expression and localization of Glut (e.g., Glut1), tumor cells exhibit enhanced glucose uptake and provide sufficient substrates for enhanced glycolysis (Ganapathy-Kanniappan and Geschwind, 2013). PI3K/Akt, HIF-1, p53, Ras and c-Myc are involved in the regulation of Glut1 (Barron et al., 2016). Moreover, there is a correlation between the overexpression of Glut1 and the poor prognosis of various tumors (Kawamura et al., 2001; Deng et al., 2018; Xiao et al., 2018). Thus, Glut has become a focal point in the field of tumor research in recent years as an important target for the gene-targeted treatment of malignant tumors. The promoting effect of Glut1 on tumor progression may be related to the enhanced glycolysis and lactate accumulation. Excess lactate then increases Rheb-GTP levels (mTORC1 activator) by inhibiting the binding of TSC2 and Rheb, ultimately activating the mTORC1 signaling pathway and tumor progression (Chen H. et al., 2021).

#### 2.2.2 Glycolytic process

The process of glycolysis is central to the energetic metabolism of tumor cells, providing most of the energy required by the cells. In this process, enzymatic reactions catabolize glucose into pyruvate. The three enzymes that are responsible for rate limitations in the glycolytic process are pyruvate kinase (PK), phosphofructokinase 1 (PFK1), and hexokinase (HK). HK2 and PKM2, the predominant isoforms of pyruvate kinase and hexokinase, respectively, are upregulated in tumor cells. These isoforms are crucial for the development and progression of tumors (Mathupala et al., 1995; Liu et al., 2016). Notably, PKM2 also possesses protein kinase activity, which, after nuclear translocation, activates gene transcription by phosphorylating histones and exerting nonmetabolic functions in tumor cells (Chen et al., 2020). For example, the PKM2/β-catenin complex is recruited to the CCND1 promoter, which encodes cyclin D1, and promotes cyclin D1 expression, which promotes tumorigenesis and tumor cell proliferation (Yang et al., 2011). In contrast to the other two key enzymes, aberrant PFK1 expression is usually not obvious in tumors. Fructose-2,6-bisphosphate, the catalytic product of PFK2, is an allosteric activator of PFK1, and tumor cells indirectly control the activity of PFK1 by upregulating PFK2 expression, which in turn promotes glycolysis (Yalcin et al., 2009; Bartrons et al., 2018). In addition, albeit infrequently, tumor cells can also enhance glycolysis by upregulating the expression or activity of noncritical enzymes, such as phosphoglycerate mutase (PGM) and phosphoglycerate kinase 1(PGK1).

#### 2.2.3 Lactate production and transport

Increased glucose intake and enhanced glycolytic processes lead to increased pyruvate production. Under normal conditions, pyruvate enters the mitochondria and can be catalyzed by the pyruvate dehydrogenase complex (PDHx) for reduction to acetyl coenzyme a, which eventually enters the tricarboxylic acid cycle for complete oxidation to carbon dioxide and water. However, in tumor cells, the rate of pyruvate production often exceeds the rate of PDHx-mediated pyruvate catabolism (Curi et al., 1988), and in some tumors, the activity of PDHx is inhibited by pyruvate dehydrogenase kinase (PDK) (Zuo et al., 2021). By upregulating lactate dehydrogenase and monocarboxylate transporter proteins, which convert pyruvate into lactate and exclude it from the cell, tumor cells ensure that their own glycolysis continues. And in this process, NADH is converted to NAD^+^, and the redox balance in tumor cells is maintained. Thus, it is easy to determine the connection between the upregulation of LDHa and MCT1/MCT4 expression levels and tumor progression (Cai et al., 2020; Zhao et al., 2018; Dell'Anno et al., 2020; Todenhöfer et al., 2018).

### 2.3 Mechanisms of enhanced glycolysis

**FIGURE 2:**
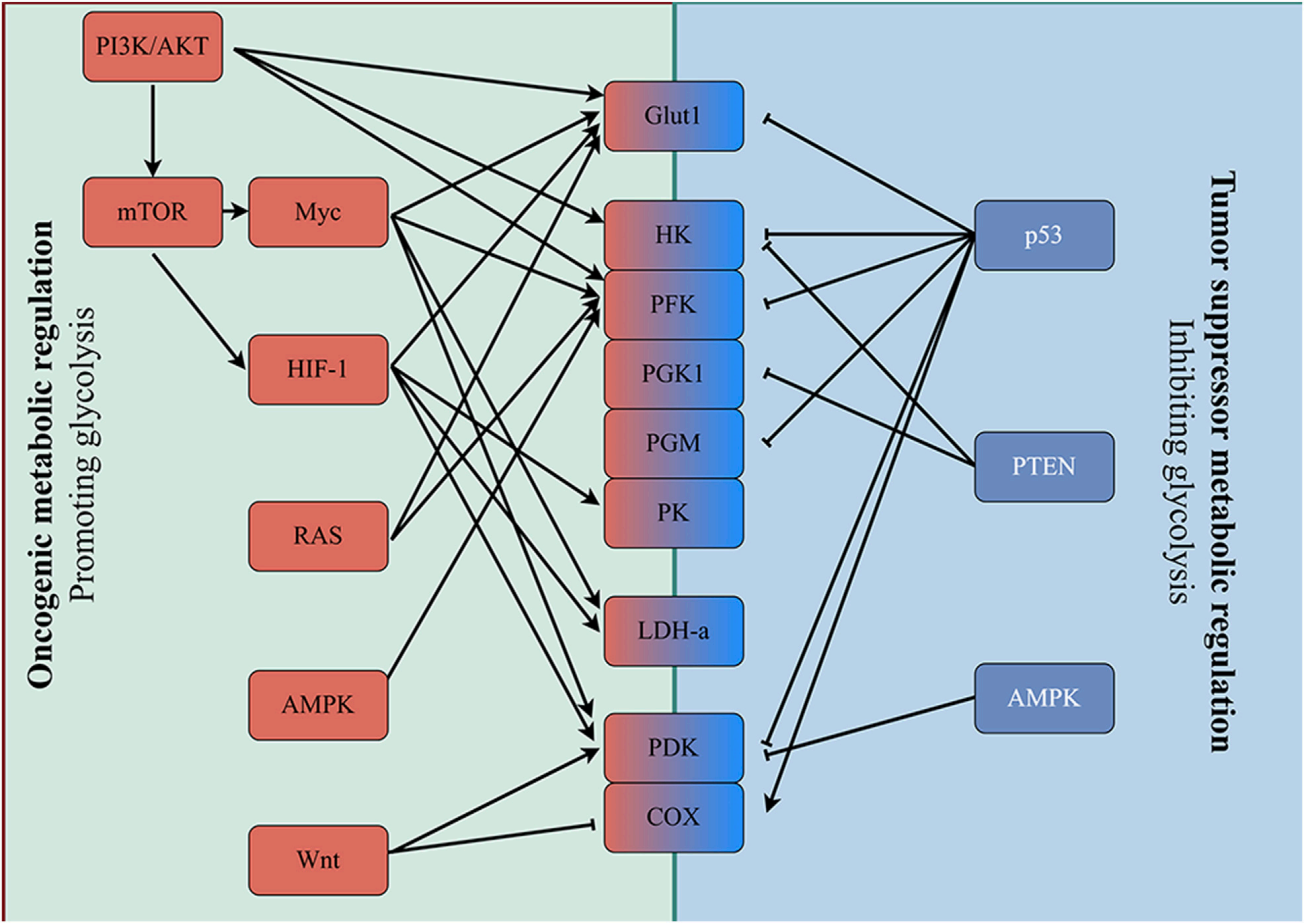
Enhanced glycolysis induced by oncogenic events. Mutation of oncogenes (e.g., RAS and MYC) and inactivation of tumor suppressor genes (e.g., p53 and PTEN) frequently occur in various tumor cells. Alterations in these genes subsequently upregulate the expression of key enzymes involved in glycolysis directly, and glycolysis is enhanced in tumor cells accordingly.

The mechanisms underlying the regulation of tumor aerobic glycolysis energy metabolism mainly include oncogenic metabolic regulation and tumor suppressor metabolic regulation (Figure 2). Oncogenic metabolic regulation mainly includes oncogenes (PI3K, MYC, Ras) and signaling pathways (Wnt). Tumor suppressor metabolic regulation mainly involves tumor suppressor genes such as PTEN and P53. Mutations in these key genes or aberrant activation of signaling pathways regulate glycolysis through a variety of pathways, including 1. Increasing the expression and the membrane localization of Glut1, which enhances the ability of the cell to take up glucose; 2. Upregulating the expression of glycolysis-related proteins or enhancing their activity, which enhances the ability of the cell to metabolize glucose into lactate; and 3. Inhibiting mitochondrial respiration, which causes cells to upregulate and depend on glycolysis. Moreover, there are also complex interactions between these genes or pathways that synergistically regulate tumor glycolysis. The details are described as follows.

#### 2.3.1 PI3K mutation

The PI3K/AKT signaling pathway is an important signaling regulatory pathway for protein synthesis, and its aberration will promote the switch of tumor metabolism to aerobic glycolysis (Edinger and Thompson, 2002; Elstrom et al., 2004). Furthermore, the enhanced glycolytic activity caused by the activation of this pathway can be observed in various solid tumors (Zhang T. et al., 2019; Feng et al., 2020; Chakraborty et al., 2022; Dong et al., 2022). When this pathway is consistently activated, it can increase the expression of Glut (Plas et al., 2001), stimulate the expression and activity of enzymes related to glycolysis (Deprez et al., 1997; Rathmell et al., 2003), and improve the capacity of tumor cells to metabolize glucose and engage in glycolysis. In addition, interactions between the ROS/PI3K/Akt pathway and the Wnt/β-catenin signaling pathway will promote the expression of HIF-1α, another factor that promotes glycolysis. The upregulation of HIF-1α expression further increases glycolytic activity, enabling tumors to develop a drug-resistant phenotype (Dong et al., 2022).

#### 2.3.2 PTEN mutation

In essence, PTEN is a protein phosphatase that functions as a tumor suppressor by breaking down phosphatidylinositol-3,4,5-trisphosphate (PIP3) and suppressing the PI3K/AKT pathway, which is also its primary mechanism for inhibiting glycolysis. The activation of AKT induced by PTEN deletion can enhance glycolysis (Zhai et al., 2022). In addition, PTEN can directly regulate glycolysis in tumors. The glycolysis-associated enzyme phosphoglycerate kinase 1 (PGK1), which has protein kinase activity, can catalyze autophosphorylation, and phosphorylated PGK1 exhibits enhanced enzyme activity. PTEN inhibits the autophosphorylation of PGK1, thereby directly inhibiting tumor glycolysis (Qian et al., 2019). Moreover, prostate cancer patients harboring PTEN/P53 mutations exhibit significantly enhanced intracellular HK2-mediated glycolysis and are resistant to castration therapy (Deng and Lu, 2015).

#### 2.3.3 p53 mutation

p53 is a very important tumor suppressor gene in the human body and its mutations are common in many types of cancer. Studies have shown that the promoters of Glut1 and Glut4 are directly regulated by p53. However, the inhibitory effect of p53 on the promoter of Glut4 is significantly stronger than that on Glut1. This is because Glut1 is a widely available glucose transporter (Schwartzenberg-Bar-Yoseph et al., 2004). Moreover, p53 can inhibit glycolysis by interfering with the transport of Glut1 to the membrane. p53 mutations promote tumorigenesis by activating the RhoA/ROCK signaling-mediated translocation of Glut1 to the plasma membrane to stimulate the “Warburg effect" (Zhang et al., 2013). PFK1 is metabolically activated by fructose-2,6-bisphosphate (F-2,6-BP), and as F-2,6-BP accumulates, it becomes more active and encourages glycolysis. TIGAR, a p53-induced protein, catalyzes the breakdown of F-2,6-BP, inhibiting PFK1 enzyme activity and glycolysis (Bensaad et al., 2006). Furthermore, by controlling the production of phosphoglycerate mutase (PGM), another glycolysis-related enzyme, p53 prevents glycolysis (Kondoh et al., 2005). Moreover, p53 can also regulate mitochondrial function and oxygen consumption and reduce glycolysis. The control of the mitochondrial cytochrome c oxidase (COX) complex depends on the p53-dependent production of cytochrome c oxidase 2 (SCO2). Deficiency in p53 leads to impairment of the mitochondrial oxidative respiratory chain and promotes the conversion of OXPHOS to glycolysis. Another study showed that p53 was able to restore colorectal cancer cell chemosensitivity to fluorouracil (5-FU) via miR-149-3p/PDK2-mediated inhibition of glycolysis (Liang et al., 2020).

#### 2.3.4 MYC mutation

MYC family genes are a group of oncogenes that are frequently and abnormally active in human cancers. Glucose metabolism genes, primarily Glut1, HK2, PFK and LDHa, are directly regulated by c-Myc (Shim et al., 1997; Osthus et al., 2000). Studies focusing on glucose metabolism inhibitors targeting c-Myc have shown that they can repress the expression of Glut1 and LDHa in cancer cells. In addition, in a hypoxic state, c-Myc interacts with HIF-1, which induces PDK1 production. This inhibits the conversion of pyruvate to acetyl-CoA catalyzed by PDH, thereby suppressing mitochondrial respiration and ultimately promoting anaerobic glycolysis (Dang et al., 2009).

#### 2.3.5 RAS mutation

RAS mutations are among the most common carcinogenesis factors (Chen J. et al., 2021). The regulation of glycolysis by RAS is common. On the one hand, the translocation of mutant RAS to mitochondria results in the abnormal opening of ion channels, impaired mitochondrial membrane potential and oxidative phosphorylation, and the cell is forced to increase its demand for glycolysis (Hu et al., 2012). On the other hand, the glycolytic fluxes of RAS mutant cells were equilibrated at higher levels by upregulating Glut1 and PFK2 expression (Yun et al., 2009; Telang et al., 2006). In addition, there is extensive cooperation between RAS and Myc and AMPK pathways in regulating glycolysis (Ying et al., 2012).

#### 2.3.6 Wnt pathway

In malignant tumors, hyperactivation of the Wnt pathway affects the regulation of proliferation, angiogenesis, migration, epithelial–mesenchymal transition (EMT), and cell survival (Parsons et al., 2021). More recently, Wnt signaling has been linked to metabolic reprogramming in cancer cells. Differently, the Wnt pathway induces compensatory enhancement of glycolysis mainly by inhibiting mitochondrial respiration. By regulating the expression of downstream mitochondria-associated genes such as cytochrome C oxidase (COX) and pyruvate dehydrogenase kinase 1 (PDK1), Wnt controls glycolysis (Lee et al., 2012; Pate et al., 2014; Zuo et al., 2021). The Wnt pathway promotes the expression of PDK1 and inhibits the expression of COX, which limits the supply of pyruvate to mitochondria and mitochondrial OXPHOS activity, respectively. This results in a compensatory increase in glycolysis to offset ATP loss. Moreover, Wnt signaling inhibits FTO-mediated downregulation of m6A on Myc mRNA and promotes the translation of Myc mRNA, which in turn regulates glycolysis, proliferation, and carcinogenesis in tumor cells via Myc.

#### 2.3.7 AMPK pathway

AMPK, a key energy state sensor and regulator of energy homeostasis, consists of catalytic α, regulatory β, and γ subunits. AMPK is involved in regulating tumor glycolysis, but the results of this regulation are inconsistent. The majority of related research points to a negative relationship between the rate of glycolysis and the activation of AMPK signaling (Bi et al., 2021). In colorectal cancer, AMPK downregulates PDK4 expression and restricts the flow of pyruvate to mitochondria for OXPHOS(Wei et al., 2023). Another study indicated that AMPK-mediated phosphorylation of the pyruvate dehydrogenase complex (PDHc) catalytic subunit (PDHA) S295 during tumor metastasis restored PDHc activity through spatial competition for inhibition of PDK-mediated suppression of PDHA S293 phosphorylation, promoted a shift in cellular metabolism from glycolysis to OXPHOS, and enhanced metabolic adaptation in metastatic cells (Cai et al., 2020). However, there is also support that AMPK activation promotes tumor glycolysis. Activated AMPK enhances PFK2 activity by phosphorylating PFK2, inducing F-2,6-BP accumulation and enhancing glycolysis (Doménech et al., 2015). This result is in line with the function of AMPK in muscle tissue (Marsin et al., 2002). The relationship between AMPK and tumor glycolysis deserves further investigation.

#### 2.3.8 HIF-1 pathway

In addition to oncogenic signals, tumor cell metabolism is regulated by the hypoxic TME. The hypoxia phenotype is intimately associated with the HIF-1 pathway. HIF-1α and HIF-1β are the two subunits that make up HIF. Intracellular HIF-1β expression is stable, whereas prolyl hydroxylase controls HIF-1α expression. Hydroxylated HIF-1α undergoes ubiquitination-mediated degradation by VHL, so the HIF-1 signaling pathway is tightly regulated. Under hypoxic conditions, prolyl hydroxylase activity is inhibited by mitochondria-generated ROS, and HIF-1α is upregulated and forms a complex with HIF-1β to act as a transcriptional regulator to regulate the transcription of downstream genes. The targets of HIF-1 include genes encoding Glut, glycolysis-related enzymes, and LDHa, and elevated expression of these genes promotes glycolysis and lactate production (Semenza et al., 1994). Furthermore, the HIF-1 signaling pathway suppresses pyruvate dehydrogenase activity, limits OXPHOS, and causes the reduction of more pyruvate to lactate by controlling the expression of pyruvate dehydrogenase kinase (Kim et al., 2006).

#### 2.3.9 mTOR pathway

The mTOR pathway, which includes mTOR complex 1 (mTORC1), mTOR complex 2 (mTORC2), and putative mTOR complex 3 (mTORC3), has important roles in mammalian metabolism and physiology (Liu and Sabatini, 2020). Because its catalytic structural domain resembles PI3K, mTOR is sometimes classified as an atypical member of the PI3K-related kinase family. However, unlike the PI3K pathway, direct regulation of glycolysis by the mTOR pathway has rarely been reported. In general, the mTOR pathway interacts extensively with other signaling pathways and in this way is involved in the regulation of tumor metabolism. For example, PI3K/AKT/mTORC1/HIF-1alpha are usually considered as a whole and are involved in the regulation of tumor glycolysis (Düvel et al., 2010). mTORC2 is also involved in the regulation of glycolysis through a different mechanism. Although it can also be activated by AKT, mTORC2 controls glycolysis by affecting the expression of c-Myc. mTORC2 regulates the expression of c-Myc by inhibiting the deacetylation of histone proteins, which ultimately regulates glycolysis (Xu et al., 2019).

## 3 What about glycolysis reprogramming in the TME?

**FIGURE 3:**
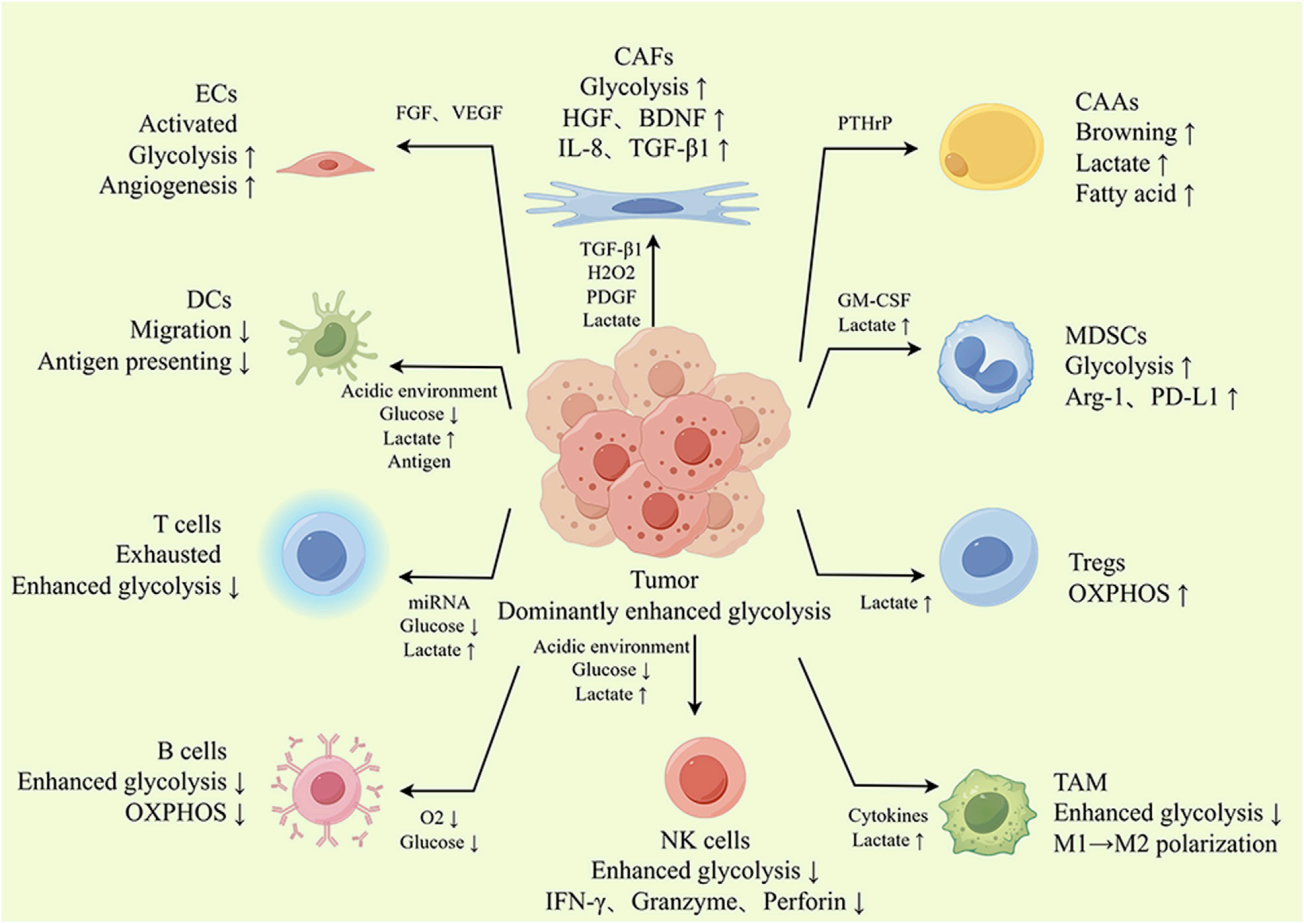
The glycolysis landscape of the TME. Tumor cells can induce a protumor phenotype in the TME by secreting metabolites and signaling molecules such as cytokines and lactate, thus further contributing to cancer progression. For example, stromal cells activated by tumor cells undergo glycolytic enhancement, and their glycolytic products can serve as an energy source for tumor cells. In addition, lactate produced by tumor cell glycolysis can also induce CAFs to produce protumor cytokines, promoting tumor growth, metastasis and drug resistance. Tumor-infiltrating T cells, B cells, DCs and NK cells also showed enhanced glycolysis. However, with powerful ability to plunder glucose, tumor cells restrict glycolysis in T cells, B cells, DCs and NK cells, which leads to dysfunction of these cells. Similarly, glycolysis is involved in regulating the polarization and functional phenotype of TAMs. Tregs take up lactate for OXPHOS, avoiding glucose competition and adapting to the environment of high lactate, which is conducive to fully exploiting their tumor-promoting effect. In summary, glycolysis promotes crosstalk between tumor cells and TME cells and alters the functional phenotype of TME cells.

The coevolutionary process involving the tumor and the TME leads to tumor growth. Many nontumor cells inside the TME experience metabolic changes akin to the “Warburg effect” as tumors advance, and they increasingly rely on glycolysis to carry out their biological tasks. This means that to fulfill their functional needs, TME cells will all have to engage in competition for limited glucose and need to learn to adapt to the high-lactate environment that results from enhanced glycolysis. This section will then concentrate on how TME cells behave differently in the face of this metabolic challenge and how they interact with tumor cells. Typically, antitumor TME cells have difficulty adapting to the hostile environment resulting from enhanced glycolysis, whereas the tumor-promoting TME component sustains enhanced glycolysis and adapts more readily to the high-lactate environment with the support of tumor cells. The details are described as follows (Figure 3).

### 3.1 Glycolysis reprogramming in stromal cells

#### 3.1.1 Cancer-associated fibroblasts

One significant stromal cell type in the TME is cancer-associated fibroblasts (CAFs). Like in tumor cells, glycolysis is enhanced in CAFs, known as the “reverse Warburg effect”. Glycolysis of CAFs is induced and permitted by tumor cells. Tumor cells hijack CAFs and reprogram their metabolism for aerobic glycolysis through multiple pathways, secreting metabolites such as pyruvate and lactate, which are taken up by cancer cells as alternative carbon sources to support their metabolic needs. *In vitro* experiments demonstrated that the glycolytic activity of CAFs was enhanced after coculture with cancer cells. Mechanistically, tumor cells produce and secrete hydrogen peroxide, which induces oxidative stress in nearby CAFs, decreasing mitochondrial activity and increasing glucose uptake (Martinez-Outschoorn et al., 2011). Additionally, cytokines found in the TME, such as PDGF and TGF-β1, are also involved in the metabolic reprogramming of CAFs. By downregulating isocitrate dehydrogenase expression in CAFs, these cytokines modify the ratio of tricarboxylic acid cycle intermediate metabolites. This inhibits prolyl hydroxylase activity and stabilizes the HIF-1 signaling pathway, which in turn promotes glycolysis in CAFs(D. Zhang et al., 2015). In addition, tumor-produced exosomes harboring the ITGB4 protein (Sung et al., 2020) or miR-105 (Yan et al., 2018) are taken up by CAFs and promote glycolysis by affecting mitochondrial autophagy or the Myc pathway in CAFs, respectively.

In some tumors, such as prostate, gastric, pancreatic, and breast cancers, CAFs are thought to be receptors for lactate in the TME. Lactate can induce stromal cells such as normal fibroblasts (Linares et al., 2022), mesenchymal stem cells (MSCs) (Bhagat et al., 2019), and even adipocytes (Andreucci et al., 2023) to acquire the CAF phenotype. As a metabolite, lactate can affect the NAD^+^/NADH balance, downregulating NAD^+^ and thus inhibiting PARP-1 activity, promoting fibroblast transition to CAFs(Linares et al., 2022). Lactate can also be converted to other metabolic intermediates, such as fumarate and succinate, through the metabolic cycle, thereby affecting the balance between fumarate, succinate and α-ketoglutarate, which ultimately affects TETase activity, causes changes in DNA methylation levels and promotes the expression of certain protumor factors in CAFs(Andreucci et al., 2023). The “Warburg effect” is particularly prominent in drug-resistant tumor cells, where lactate production also promotes the secretion of BDNF(Jin et al., 2021) and HGF (Bhagat et al., 2019) from CAFs, which are molecules that promote drug tolerance.

#### 3.1.2 Endothelial cells

Endothelial cells (ECs) are ubiquitous in tumors and are necessary for vascular development and function, providing oxygen and nutrients for tumor growth and participating in tumor metastasis (Ghiabi et al., 2014). Although ECs are located in the inner layer of blood vessels and lymphatic vessels and have easy access to oxygen, activated ECs are highly dependent on glycolysis (De Bock et al., 2013). The glycolysis dependence of ECs in tumor vessels is even greater than that of ECs in the normal activated state, accompanied by enhanced expression of Glut1 and HK2(Cantelmo et al., 2016). Several signals in the TME, including hypoxia and proinflammatory cytokines (Yu et al., 2017) or hormonal signals (Trenti et al., 2017), can upregulate glycolysis in ECs. By activating the PI3K/AKT pathway, VEGF (vascular endothelial growth factor) released by tumor cells enhances Glut1 expression in ECs(Yeh et al., 2008). Indeed, the expression of genes associated with glycolysis is frequently upregulated in ECs, and PFK2 is a crucial glycolysis regulating gene. Downregulation of PFK2 expression in ECs normalizes the tumor vasculature, which can effectively reduce tumor metastasis and enhance the effect of chemotherapy (Cantelmo et al., 2016).

In addition, the glycolysis product lactate can regulate the function of ECs through a variety of pathways. First, lactate acts as a signaling molecule to enhance VEGF signaling and stimulate angiogenesis by activating the HIF-1 (Hunt et al., 2007) and PI3K/AKT pathways (Hunt et al., 2007). Second, lactate, a metabolic substrate, is taken up by ECs to activate the autocrine NF-κB/IL-8 (CXCL8) pathway by upregulating intracellular ROS levels, driving cell migration and tubule formation (Végran et al., 2011). Finally, the acidic tumor microenvironment induced by lactate accumulation induces endoplasmic reticulum stress in endothelial cells via the proton-sensing G protein-coupled receptor GPR4, which regulates vascular growth and inflammatory responses in the acidic microenvironment (Dong et al., 2017).

#### 3.1.3 Adipocytes

Adipocytes are present in many tissues, including tumor tissues. Tumor-associated adipocytes (TAAs) are an important part of the TME (Pallegar and Christian, 2020). Like in CAFs, there is an active exchange of metabolites between adipocytes and tumor cells. Upon coculture with tumor cells, adipocytes exhibit a characteristic cellular morphology and phenotype similar to that of fibroblasts, accompanied by increased lactate secretion (Simiczyjew et al., 2023). Adipocytes tend to enhance glycolysis in the TME, as in renal clear cell carcinoma, where the browning of adipocytes is activated by parathyroid hormone-related proteins secreted by cancer cells, leading to the production of excess lactate (Wei et al., 2021), which is taken up and utilized by cancer cells as an energy source (Micallef et al., 2021). In addition, there is positive feedback between the glycolytic product lactate and adipocytes that promotes tumor progression. In a high-lactate environment, adipocytes can dedifferentiate toward myofibroblasts and secrete more free fatty acids, among other factors, to exert their tumor-promoting ability (Andreucci et al., 2023). Lactate has been reported to be taken up by adipocytes as a raw material for fat synthesis (Saggerson et al., 1988) or to promote fat synthesis via the lactate-GPR81 axis (Ahmed et al., 2010) and ultimately benefit tumor cells.

### 3.2 Glycolysis reprogramming in immune cells

Typically, glycolytic remodeling also occurs in immune cells in the TME. With the exception of Treg cells, which are relatively flexible in metabolism, all other immune cells use glycolysis as the primary mode of sugar metabolism in their active state. However, tumor cells promote glycolysis in cells associated with tumor immunosuppression through various mechanisms, whereas antitumor immune cells need to compete with tumor cells for glucose to maintain glycolysis. In addition, immune cells that exert antitumor effects seem to have difficulties adapting to the high-lactate TME.

#### 3.2.1 Tumor-infiltrating lymphocytes

Tumor-infiltrating lymphocytes (TILs) are important members of the immune surveillance system for tumors and are mainly divided into CD8^+^ T cells and CD4^+^ helper T cells. Target cells are eliminated by CD8^+^ T lymphocytes via FASL-FAS-mediated cell death or granzyme- and perforin-mediated apoptosis. Unfortunately, CD8^+^ T lymphocytes are typically impaired or diminished in malignancies (Philip and Schietinger, 2022). The functions of CD4^+^ T cells in malignancies are dual. Th1 CD4^+^ T cells directly kill cancer cells by producing interferon gamma (IFN-γ) and TNF-α, and they also assist cytotoxic CD8^+^ T cells and B cells in their antitumor activities. However, CD4^+^ T cells of the Th2 subtype release anti-inflammatory molecules that have tumor-promoting properties (Borst et al., 2018).

One indicator of the activation of CD8^+^ T cells is the transition from OXPHOS to aerobic glycolysis, and the mTORC1-HIF-1 pathway is critical for mediating this metabolic transition (Finlay et al., 2012; Kirchmair et al., 2023). The expression of glycolytic proteins and the rate of glycolysis were greater in differentiated CD8^+^ effector T cells than in naïve T cells. Increased aerobic glycolysis is essential for promoting the production of biosynthetic precursors required for the proliferation of effector T cells and the production of effector molecules such as IFN-γ, IL-2, IL-17 and granzyme B in T cells. When glycolysis in CD8^+^ effector T cells is restricted, dysfunction results (Cham and Gajewski, 2005). Research indicates that after the inhibition of glycolysis through glucose deprivation or 2-deoxy-D-glucose (2-DG), the expression of genes induced by TCR/CD28 signaling in CD8^+^ effector T cells is partially inhibited, which includes the expression of IFN-γ(Cham et al., 2008). Mechanistically, the glycolysis-associated enzyme GAPDH acts as an mRNA-binding protein to bind to IFN-γ mRNA and limit its translation when glycolysis is inhibited in CD8^+^ effector T cells (Chang et al., 2013). Limiting glucose intake inhibits CD4^+^ T cells’ ability to perform immunological surveillance because activated CD4^+^ T cells are likewise dependent on glycolysis (Michalek et al., 2011). The glycolytic intermediate phosphoenolpyruvate safeguards the normal antitumor function of Th1 CD4^+^ T cells by inhibiting Serca-mediated endoplasmic reticulum calcium uptake activity and maintaining intracellular calcium flux and Ca_2_^+^-NFAT1 pathway activation (Ho et al., 2015). Ensuring the supply of glucose and maintaining the level of glycolysis are essential for the antitumor immune effects of tumor-infiltrating lymphocytes. However, in the TME, T cells are at a disadvantage in terms of glucose competition. In addition, tumor cells encourage effector T cells to substantially express miR-26a and miR-101 by controlling glucose in the TME, which leads to a decrease in EZH2 expression and dysfunction of effector T cells, including decreased glycolysis (Zhao et al., 2016).

In addition, lactate accumulation in the TME has a negative impact on the function of T cells. It has been reported that the lactate concentration, which is inversely related to the degree of CD8^+^ T-cell infiltration, can be used to predict immunological function in breast cancer patients (Yin et al., 2022). Lactate exposure significantly reduces IFN-γ production in a dose-dependent manner in CD8^+^ T cells (Huang et al., 2023). Succinate autocrine acts on CD8^+^ T cells via the SUCNR1 receptor to maintain their function. Lactate can affect the metabolism of CD8^+^ T cells and inhibit pyruvate carboxylase activity, thereby reducing the production and autocrine secretion of succinate and inhibiting the cytotoxic activity of CD8^+^ T cells (Elia et al., 2022). Lactate produced by tumor cells increases the susceptibility of tumor-specific cytotoxic CD8^+^ T lymphocytes to activation-induced cell death via NF-κB inactivation, and blocking lactate production can overcome tumor resistance to immunotherapy and improve therapeutic effects (Liu et al., 2023).

#### 3.2.2 B cells

The main humoral immune mediators are B lymphocytes. Through complement activation and antibody-dependent cytotoxicity, B cells in malignancies produce anticancer effects (Laumont et al., 2022). In contrast to other cells, activated B cells exhibit enhanced both OXPHOS and glycolysis (Blair et al., 2012; Caro-Maldonado et al., 2014). However, the “best-of-both-worlds” metabolic strategy of B cells does not give them a metabolic advantage because the suppression of either glycolysis or oxidative phosphorylation can inhibit the growth and function of B cells (Diaz-Muñoz et al., 2015; Jayachandran et al., 2018). Even worse, the low-glucose/low-oxygen TME may concurrently block glycolysis and OXPHOS in B cells. However, more information needs to be obtained, as our knowledge of the metabolism of B cells in malignancies is still lacking.

#### 3.2.3 Tregs

Regulatory T cells (Tregs), which are the guardians of immunological homeostasis, are a highly immunosuppressive fraction of CD4^+^ T cells. In cancer, Tregs suppress antitumor immunity through different mechanisms. Tregs are less dependent on glycolysis and rely on oxidative phosphorylation for energy production (Gerriets et al., 2015).

Foxp3 is a signature gene of Tregs. Moreover, Foxp3 represses Myc gene expression and shuts down glycolysis, inducing OXPHOS in Tregs (Angelin et al., 2017). Indeed, Tregs take up lactate as their main energy substance, and the uptake process is mediated by MCT1. Lactate enters Tregs and is converted to pyruvate to enter the tricarboxylic acid cycle, where part of it is metabolized to produce energy and the other part is converted to phosphoenolpyruvate via the gluconeogenesis pathway, which is used to replenish upstream glycolytic intermediates (Watson et al., 2021). Thus, instead of limiting Treg function, the low-glucose/high-lactate TME confers a metabolic advantage to Treg cells because of lactate accumulation. Additionally, the acidic milieu produced by lactic acid—rather than lactate itself—helps CD4^+^ T cells differentiate into Tregs when stimulated by TGF-β(Rao et al., 2023). In addition, lactate-derived phosphoenolpyruvate modulates intracellular calcium ion levels and promotes NFAT1 translocation into the nucleus, thereby upregulating the expression of PD-1 in Treg cells by affecting the Ca_2_^+^-NFAT1 pathway (Cham and Gajewski, 2005).

#### 3.2.4 Tumor-associated macrophages

Another crucial element of the TME is tumor-associated macrophages (TAMs), which are mainly divided into antitumor M1 macrophages and protumor M2 macrophages (DeNardo and Ruffell, 2019). M1 macrophages exhibit “Warburg effect"-like alterations, including increased glucose consumption and lactate release and decreased oxygen consumption. Whereas IL-4 and IL-13 induce another activation program in macrophages, macrophages (M2) activated in this mode mainly adopt the metabolic pathway of OXPHOS(Zhu et al., 2015).

In the early stages of cancer, TAMs exhibit a proinflammatory M1 phenotype to exert antitumor effects. However, these cells eventually transform into an immunosuppressive and angiogenesis-promoting M2 phenotype, which promotes tumor growth and evasion of immune surveillance (Yeung et al., 2015). There are many reasons for the phenotypic shift of macrophages, such as specific cytokines produced by tumor cells (Soki et al., 2014), hypoxia (Leblond et al., 2016) and glycolysis (Zhang D. et al., 2019; Jiang et al., 2022), and more recent studies have suggested that progressively accumulating lactate may also play a role in this process. Lactate-derived histone lysine lactylation is a novel posttranslational modification that directly stimulates the transcription of modified genes. Enhanced glycolysis in M1 macrophages leads to the accumulation of the metabolite lactate, causing the transcriptional start regions of homeostasis-associated genes (the signature genes of the M2 phenotype) to undergo increased histone lactylation and encouraging the production of these homeostasis-associated genes (Zhang T. et al., 2019). Furthermore, the expression of the macrophage-specific vacuolar ATPase subunit (ATP6V0d2), which is facilitated by TFEB, was suppressed by lactate-activated mTORC1 signaling. This prevents HIF-2α from being degraded by lysosomes and guarantees the expression of the HIF-2α downstream gene VEGF, which promotes tumor angiogenesis (Liu et al., 2019). In addition, other studies have indicated that lactate inhibits the production of inflammatory factors by activating the receptor GPR81 on the surface of macrophages. Lactate/GPR81 activates downstream AMKP and LATS signaling, which phosphorylate YAP, resulting in the retention of YAP in the cytoplasm without further stimulation of NF-κB activation or TNF-α production (Yang K. et al., 2020).

#### 3.2.5 Dendritic cells

As antigen-presenting cells that initiate and control adaptive immune responses by ensnaring antigens in infections or tumors and presenting them to T cells, dendritic cells (DCs) serve as a link between innate and adaptive immunity. The process of TLR agonist-induced metabolic switching in DCs is responsible for its activation and involves a shift from OXPHOS to aerobic glycolysis.

Further studies showed that the glycolytic process in activated DCs was activated and maintained by different mechanisms at different stages. In the early phase after receiving an effective stimulus, the activation of glycolysis is more regulated by the TBK1/IKKε/Akt pathway (Everts et al., 2014). In the early stages, the mitochondrial activity of DCs is not significantly inhibited; instead, glycolysis supplies pyruvate for mitochondrial OXPHOS. This finding also suggested that decoupling the processes of glycolysis and lactate production might be a therapeutic strategy given the adverse effects of lactate on tumor therapy. Long-term sustained increases in glycolysis rely on iNOS-mediated NO production, which damages mitochondria. After exposure to TLR agonists for a period of time, unlike the symbiosis of glycolysis and OXPHOS in the early phase, activated DCs showed increased iNOS-mediated NO production accompanied by impaired mitochondrial function and OXPHOS, and the cells relied heavily on glycolysis for ATP production, at which time the activation of DCs was impaired if glycolysis was restricted (Everts et al., 2012).

DCs capture tumor-specific antigens and subsequently transfer to local lymph nodes, where they present antigens to T cells, leading to the initiation of effector T-cell responses against tumor-specific antigens. Studies have shown that glycolysis is required for the motility and migration of DCs. Under glucose-restricted conditions, DCs exhibit significantly reduced motility, and the cellular morphology becomes rounder due to dendritic constriction, which is detrimental for DCs to migrate to localized lymph nodes (Guak et al., 2018).

In addition, lactate in the TME can also negatively affect the activation and functional realization of DCs through multiple pathways. Lactate activates the GPR81 receptor on the surface of DCs, which on the one hand inhibits cell-surface MHC2 presentation and prevents tumor-specific antigen presentation (Brown et al., 2020) and, on the other hand, affects intracellular free calcium levels, limiting the production of type I interferon through calcium/calcineurin phosphatase (CALN) signaling. Activated DCs are dependent on glycolysis, and lactate from the TME entering DCs via MCTs can negatively regulate glycolysis via feedback (Raychaudhuri et al., 2019). Lactate inhibits TLR3 and its downstream IFN-I and interferon gene-stimulating factor (STING), accelerating antigenic degradation and affecting the cross-presentation capacity of DCs (Caronni et al., 2018). In addition, some reports emphasize that lactic acid (but not lactate) impairs the viability and ability of DCs to produce type I interferon (Monti et al., 2020). The acidic environment brought about by lactic acid affects the stabilization of the antigen-MHC-I complex and is not conducive to antigen presentation (Burgdorf et al., 2020). Moreover, the lysosomal pathway is thought to be the major pathway for antigen processing and presentation in DCs, whereas lysosomal function can be modulated by lactate (Trombetta et al., 2003; Brisson et al., 2016). In addition, lactate also enhances tryptophan metabolism and kynurenine production in DCs, thereby promoting Treg induction and indirectly exerting an immunosuppressive effect (Raychaudhuri et al., 2019). C-C chemokine receptor type 7 (CCR7) facilitates the migration of DCs to adjacent lymph nodes, and lactate exposure downregulates the expression of CCR7, which affects the translocation of DCs to the local lymph and interferes with the normal process of antigen presentation (Sangsuwan et al., 2020).

#### 3.2.6 NK cells

NK cells are innate lymphoid-like cells with cytotoxicity, and the initiation of their cytotoxic function does not depend on antigen presentation; this ability allows NK cells to play an essential role in the early stage of the fight against cancer, with potent antitumor and tumor metastasis inhibition capabilities.

In the context of MCMV infection, TNFα-TNFR2 signaling upregulates NK cell glycolysis, and IL-8 contributes to this process in a complementary way by promoting TNFR2 expression upregulation. Although the study context was infection, TNFα and IL-8 are also common signaling molecules in the TME (Khan et al., 2023). After receiving effective stimulation, NK cells undergo rapid enhancement of glycolysis, and this process precedes that of CD8^+^ T cells. Upon inhibition of glycolysis, NK cells are not able to exert their antitumor effects properly (Sheppard et al., 2021). Unfortunately, because of the powerful glucose uptake by tumor cells, NK cell glycolysis is likely to be inhibited.

In addition, lactate produced by tumor cell metabolism can also inhibit the antitumor activity of NK cells through multiple pathways. Lactate disrupts NAD^+^ homeostasis in NK cells by inhibiting NAMPT transcription, disrupting the antitumor activity and capacity of NK cells (Guo et al., 2023). The acidic TME prevents NK cells from expressing perforin, granzyme, and NKp46, thereby impeding their full cytotoxic function. Lactate also inhibits the production of IFN-γ by affecting the nuclear factor of activated T cells (NFAT) in NK cells, causing acidification and disturbed energy metabolism in NK cells (Brand et al., 2016), and an acid‒base imbalance in NK cells can lead to mitochondrial stress and apoptosis (Harmon et al., 2019). Lactate can also indirectly inhibit NK cell function by promoting the infiltration of MDSCs. Fortunately, NK cells also fight the adverse effects of lactate. Lactate-treated NK cells upregulate CaMKK2 expression, which contributes to the maintenance of NK cell viability and proliferation (Juras et al., 2023).

#### 3.2.7 Myeloid-derived suppressor cells

Myeloid-derived suppressor cells (MDSCs) are considered to be pathologically activated monocytes and neutrophils and are therefore divided into two main categories: mononuclear MDSCs (M-MDSCs) and polymorphonuclear MDSCs (PMN-MDSCs). MDSCs inhibit the activity of antitumor immune cells through paracrine signaling or intercellular contact mechanism and are crucial in forming the immunosuppressive milieu (Veglia et al., 2021).

Glycolysis was also upregulated in activated MDSCs. Similar to that in CAFs, the activation of glycolysis in MDSCs occurs in response to tumor cell regulation. When stimulated by tumor-derived factors, MDSCs upregulate glycolysis-related genes and exhibit higher glycolysis rates than normal cells, which contributes to the expansion of MDSCs in hosts (Jian et al., 2017). Mechanistically, the glycolytic intermediate phosphoenolpyruvate is a potent antioxidant that prevents the apoptosis induced by excessive accumulation of ROS. High glycolysis is also a way in which MDCSs exert immunosuppressive effects. By producing excess lactate, MDSCs induce lactate accumulation within CD4^+^ T cells, resulting in the inhibition of glycolysis and other cellular functions by impairing NAD^+^ cycling (Goldmann and Medina, 2023). Inhibiting other glycolysis-dependent cells through enhanced self-glycolysis is similar to the modus operandi of tumor cells.

In addition, the glycolysis product lactate induces MDSC amplification and activation in tissues and enhances tissue immunosuppression. The amplification of immunosuppressive MDSCs is greatly increased when lactate is added to the MDSC culture system (Goldmann and Medina, 2023). Through the GPR81/mTOR/HIF-1a/STAT3 pathway, lactate stimulates the activity of MDSCs, leading to increased expression of genes that promote tumor growth and immunosuppressive effects on T cells (Yang X. et al., 2020). Lactate interacts with intracellular c-Jun and protects it from FBW7-mediated degradation, thereby promoting MDSC differentiation toward an immunosuppressive phenotype (Zhao et al., 2022).

## 4 Integrated tumor therapy strategies targeting glycolysis

A characteristic of malignancy is enhanced glycolysis, which promotes tumor development (Li et al., 2023), invasion, immunological escape, and treatment resistance. For example, in pancreatic cancer, overexpression of FOXD1 enhances Glut1 expression and promotes tumor cell proliferation, invasion and metastasis in an aerobic glycolysis-dependent manner (Cai et al., 2022). In a variety of tumor cells, Glut1-dependent enhancement of glycolysis was shown to counteract CTL-mediated cytotoxic effects by reducing ROS production and downregulating c-FLIP, a key inhibitor of TNF-α-induced cell death, and targeting Glut1 contributed to the improvement of tumor immune infiltration and sensitivity to ICB therapy (Wu et al., 2023). A study in glioblastoma also supported the negative impact of glycolysis on tumor immunity. Enhanced glycolysis facilitates tumor immune escape by promoting IκBα phosphorylation degradation and NF-κB activation-dependent induction of PD-L1 expression in an HK2-dependent manner (Guo et al., 2022). Furthermore, enhanced glycolysis is strongly associated with the acquisition of drug-resistant phenotypes in tumors (Chen et al., 2022a; Wang et al., 2023).

In brief, increased glycolysis is a poor prognostic feature that is important for several aspects of tumor development, metastasis, drug resistance, and immune evasion. Conversely, therapeutic regimens targeting glycolysis are expected to inhibit tumor progression in multiple ways. Some of the integrated tumor therapy strategies targeting glycolysis are summarized below.

### 4.1 Tumor therapeutic strategies targeting glycolysis

**FIGURE 4:**
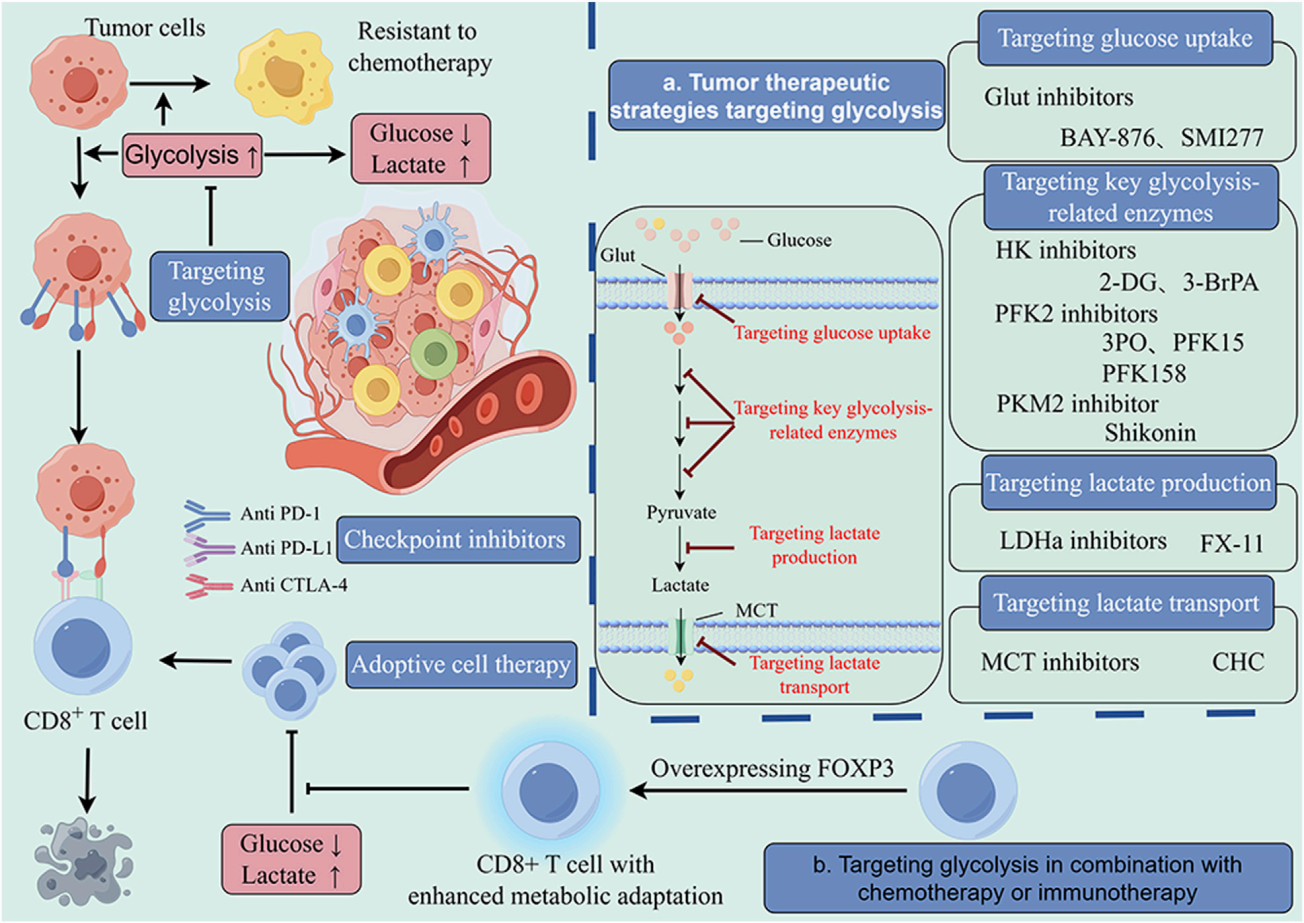
Glycolysis-targeted therapy combined with chemotherapy and immunotherapy. The enhancement of intratumor glycolysis not only promotes tumor progression and drug resistance but also plays a crucial role in the formation of immunosuppressive microenvironments. This provides a theoretical basis for a comprehensive treatment strategy targeting tumor glycolysis. In a large number of preclinical studies, glycolysis inhibitors combined with chemotherapy and immunotherapy have shown significant antitumor effects that are superior to those of single-target therapy. The main targets of these inhibitors include glucose uptake (Glut1), key glycolytic enzymes (HK, FPK, PK) and lactate production and transport enzymes (LDH, MCT). These findings provide valuable insights for optimizing current therapeutic strategies.

As mentioned earlier, glycolysis can be divided into three major steps, namely, glucose uptake, glycolytic process, and lactate production and transport, and key enzymes or molecules involved in these steps have become potential targets for cancer treatment (Figure 4).

#### 4.1.1 Targeting glucose uptake

Glut mediates the cellular uptake of glucose, and tumor cells often upregulate Glut1 expression to ensure adequate glycolytic feedstock. BAY-876 and SMI277 are drugs that target Glut with *in vivo* experimental support (Wu et al., 2020; Chen et al., 2022b). After intraperitoneal injection of Glut1 inhibitors, tumor growth was significantly limited, and tumor size was reduced. Interestingly, according to flow cytometry reports, the use of SMI277 also increased the activity of antitumor immune cells in tumor tissue. This is clearly not due to the direct action of Glut1 inhibitors because GLUT1 inhibitors similarly inhibit glycolytic processes in glycolysis-dependent antitumor immune cells, thereby inhibiting the normal function of these cells as well (Chen et al., 2023). It is suspected that such glycolysis inhibitors, by decreasing the amount of glucose that tumor cells take up, relatively alleviate the competitive pressure for glucose in tumor tissues, improving immune cells’ access to glucose, which may lead to an increase in the quantity and degree of activation of antitumor immune cells, such as CD8^+^ T cells.

#### 4.1.2 Targeting key glycolysis-related enzymes

Hexokinase (HK), phosphofructokinase 1 (PFK1), and pyruvate kinase (PK) are the three rate-limiting enzymes of the glycolytic process. Hexokinase catalyzes the generation of glucose-6-phosphate from glucose, the first step of the glycolytic reaction, and 2-DG and 3-BrPA specifically inhibit the enzymatic activity of HK. Numerous studies have demonstrated the stable antitumor capacity of 2-DG and 3-BrPA, and neither drug has toxic effects on normal cells in other organs at therapeutic doses (Fan et al., 2019; Pajak et al., 2019). PFK1 is the second restricting enzyme in glycolysis, and its activity is regulated by the catalytic product of PFK2. PFK2 phosphorylates fructose-6-phosphate to produce F-2,6-BP, a metabolic activator of PFK1, and significantly increases the catalytic activity of PFK1. *In vivo* and *ex vivo* experiments have demonstrated that 3PO, PFK15, and PFK158 exert antitumor effects by inhibiting the activity of PFKFB3, a common isoform of PFK2(Kotowski et al., 2021). PKM2 is the last rate-limiting enzyme of glycolysis, and shikonin can inhibit tumor growth in mice by inhibiting PKM2-mediated aerobic glycolysis (Zhao et al., 2018).

#### 4.1.3 Targeting lactate production and transport

LDHa catalyzes the formation of lactate from pyruvate, a process that produces NAD^+^ that is reused to maintain glycolysis. FX-11 inhibits LDHa and is a reliable and safe therapeutic regimen for LDHa-dependent tumors (Le et al., 2010). MCTs are responsible for transporting lactate out of the cell to avoid product accumulation and intracellular acidification. Studies have shown that knockdown of MCT1 inhibits cancer cell proliferation and migration, thereby suppressing tumor progression (Li et al., 2021). There are many inhibitors of MCT, but few have been tested *in vivo*. CHC was shown to inhibit the MCT1-induced switch from OXPHOS to glycolysis, effectively slowing tumor growth in mice (Sonveaux et al., 2008).

There are also many drugs targeting glycolysis that are not listed, and their antitumor effects have only been demonstrated *in vitro*, with a lack of *in vivo* experimental evidence to support them. Considering that normal cells also undergo glycolysis, the toxic effects of these drugs, which act by inhibiting glycolysis, on the whole organism should be investigated.

### 4.2 Targeting glycolysis in combination with chemotherapy

Malignant tumors usually rely on aerobic glycolysis for rapid growth and chemotherapy resistance. Compared with oxaliplatin-sensitive cells, oxaliplatin-resistant colorectal cancer cells exhibit upregulated expression levels of PKM2, a key enzyme in glycolysis, and increased levels of glycolysis. By the uptake of exosomes containing the circRNA hsa_circ_0005963 (which acts as a sponge for miR-122) secreted by drug-resistant cells, PKM2-mediated enhancement of glycolysis and drug resistance was also observed in sensitive cells (Wang et al., 2020). In glioblastoma (GBM), HK2 expression is correlated with chemoresistance, and HK2 deficiency increases glioblastoma susceptibility to temozolomide (TMZ) (Zhang et al., 2020). Furthermore, in estrogen receptor-positive breast cancer, PKM2-dependent upregulation of glycolysis leads to decreased adriamycin sensitivity in tumors. However, chemosensitivity was restored after treatment with the glycolysis inhibitor 2-DG (Qian et al., 2018). Another study reported that LDHa activity and expression were upregulated in adriamycin-resistant osteosarcoma cells and that the resistant cells exhibited highly activated glycolysis and were more dependent on the glucose supply. *In vitro* and *in vivo*, the combination of adriamycin and the glycolysis inhibitor oxamate has synergistic effects on the treatment of osteosarcoma (Hua et al., 2014). These findings show that glycolysis plays a major role in chemotherapy resistance, and the combination of glycolysis inhibitors and chemotherapeutic agents might increase the effectiveness of tumor chemotherapy. According to recent studies, the targeted delivery of the chemotherapeutic agents HCPT and siMCT-4 to tumors with the help of silica nanoparticles effectively suppressed tumor growth through chemotherapy and the inhibition of lactate efflux, and this approach restored the tumor-associated macrophage (TAM) M1 phenotype and CD8^+^ T-cell activity *in vivo*, which greatly improved the tumor immune microenvironment (Li et al., 2020).

### 4.3 Targeting glycolysis in combination with immunotherapy

Cancer immunotherapies include checkpoint inhibitors (ICIs) and adoptive cell therapy (ACT). ACT enhances antitumor immunity by directly delivering therapeutically modified immune cells. In contrast, immune checkpoint inhibitors are essentially a class of monoclonal antibodies that target key proteins of the immunosuppressive pathway called checkpoints, such as programmed cell death protein 1 (PD-1), cytotoxic T lymphocyte-associated protein 4 (CTLA-4), and programmed death ligand 1 (PD-L1).

Overexpression of glycolysis-related proteins leads tumor cells to tolerate T-cell-mediated cytotoxic effects, while inhibiting glycolysis enhances T-cell-mediated antitumor immunity (Cascone et al., 2018). Higher lactate levels in the TME hinder the proliferation and generation of cytokines in T cells. Therefore, in preclinical studies, attempts were made to combine bicarbonate (neutralizing lactic acid) with anti-CTLA-4, anti-PD-1 or ACT, a strategy that greatly improved the efficacy of tumor immunotherapy (Pilon-Thomas et al., 2016). Enhancing the metabolic adaptation of T cells in nutrient-competitive TMEs is also a strategy for improving the efficacy of ACT. As previously described, FOXP3 promotes the aerobic oxidation of Treg cells by facilitating lactate uptake, which greatly enhances the adaptability of Treg cells. It has been shown that overexpression of FOXP3 also enhances the metabolic adaptation of CD8^+^ T cells, which facilitates their tumor recruitment, proliferation and cytotoxicity and improves the therapeutic efficacy of ACT (Conde et al., 2023). Recent data indicate that *in situ* injection of the thermogel Gel@B-B containing the Glut1 inhibitor BAY-876 and a blocker of PD-1/PD-L1 BMS-1 significantly delays tumor growth, prolongs survival time and improves ICB treatment outcomes in an *in situ* mouse model of GBM by dual modulation of metabolism and immunity in glioblastoma (GBM) (Li et al., 2024).

## 5 Conclusion and outlook

Enhanced glycolysis is a hallmark of malignancy characterized by a generalized enhancement of glucose uptake, glycolytic process, and lactate production and transport, which influences tumor progression, invasion, immune escape, and drug resistance. Mutations in key genes or aberrant activation of signaling pathways are important drivers that promote enhanced glycolysis in tumor cells. Metabolic shifts in tumor cells further modify the TME and affect the glycolysis and the functional phenotype of TME cells. Through the establishment of glycolytic dominance, for example, tumor cells inhibit glycolysis and the function of antitumor immune cells, such as CD8^+^ T cells, DCs, and NK cells, to form a tumor-immunosuppressive microenvironment, which ultimately promotes tumor progression and leads to tumor immune escape and therapeutic resistance. Therefore, targeting glycolysis is a promising multidimensional therapeutic approach. However, drugs targeting the glycolytic pathway are still in the preclinical stage, despite strong animal experimental data supporting the enhanced antitumor effect of glycolysis inhibitors when combined with immunotherapy or chemotherapy. Owing to the complexity of metabolism, there is still much remaining to be done in the development of efficient medications that target tumor glycolysis.
